# New Appliances and Things Medical

**Published:** 1896-06-27

**Authors:** 


### NEW APPLIANCES AND THINGS MEDICAL.

### LIQUOR FERRO-MANGANI PEPTONATI, AND LIQUOR FERRO-MANGANI SUCCHARATI.

(Eugen Dieterich, Helfenberg; London Agent, M. Buchner, 149, Houndsditch, London, E.C)

The value of manganese either alone or in combination with iron is gradually forcing itself upon the attention of medical men for the treatment of cases of anæmia, chlorosis, and defective assimilation. Although most of us are familiar with the hypophosphite, phosphate, sulphate, and oxide of this element, they prove, in practice, difficult of administration either because of their insolubility or because of their astringent qualities. These new preparations of the metal, presented as they are in the form of peptonates or saccharates combined with iron, possess no styptic or astringent effects, and, further, can be more readily assimilated than can the inorganic salts of the same metals. The preparations, which have been the outcome of patient research in a laboratory where every facility is offered for careful work of this nature, will be gladly accepted by medical men in England in place of the disagreeable and inelegant mixtures which are the result of individual prescriptions.

